# Quantification of Hepatorenal Index for Computer-Aided Fatty Liver Classification with Self-Organizing Map and Fuzzy Stretching from Ultrasonography

**DOI:** 10.1155/2015/535894

**Published:** 2015-07-13

**Authors:** Kwang Baek Kim, Chang Won Kim

**Affiliations:** ^1^Department of Computer Engineering, Silla University, Busan 617-736, Republic of Korea; ^2^Department of Radiology, School of Medicine, Pusan National University, Pusan National University Hospital, Busan 602-739, Republic of Korea

## Abstract

Accurate measures of liver fat content are essential for investigating hepatic steatosis. For a noninvasive inexpensive ultrasonographic analysis, it is necessary to validate the quantitative assessment of liver fat content so that fully automated reliable computer-aided software can assist medical practitioners without any operator subjectivity. In this study, we attempt to quantify the hepatorenal index difference between the liver and the kidney with respect to the multiple severity status of hepatic steatosis. In order to do this, a series of carefully designed image processing techniques, including fuzzy stretching and edge tracking, are applied to extract regions of interest. Then, an unsupervised neural learning algorithm, the self-organizing map, is designed to establish characteristic clusters from the image, and the distribution of the hepatorenal index values with respect to the different levels of the fatty liver status is experimentally verified to estimate the differences in the distribution of the hepatorenal index. Such findings will be useful in building reliable computer-aided diagnostic software if combined with a good set of other characteristic feature sets and powerful machine learning classifiers in the future.

## 1. Introduction

Fatty liver or hepatic steatosis is a common histologic finding in human liver biopsy specimens, and nonalcoholic fatty liver disease (NAFLD) is the most common cause of fatty liver [1]. The prevalence of NAFLD in the general population is estimated to be 20–30% in Western countries [2], but this number is considerably higher in people with type 2 diabetes or obesity [3] and recent report found a similar tendency in Asian countries as well [4]. In a majority of patients, NAFLD is associated with metabolic risk factors such as obesity, diabetes mellitus, and dyslipidemia [5]. NAFLD is not only a liver disease but also an early mediator that reflects a metabolic disorder [6].

Liver biopsy is the gold standard for the quantification of hepatic steatosis. However, it is difficult for most patients to accept it due to its invasiveness and a significant degree of sampling error [7].

Ultrasonography (US) is an appealing technique compared with computed tomography (CT) and magnetic resonance imaging (MRI) in detecting the fatty infiltration of the liver because of its simplicity, low cost, noninvasive nature, and widespread availability.

However, almost always, the use of US methodologies in diagnosis suffers from several limitations including operator dependency, subjective evaluation, and limited ability to quantify the amount of fatty infiltration, and, ultimately, it is frequently regarded as being unable to provide an accurate measurement of the liver fat content [8].

Recently, there has been notable progress in US methodologies. The overall sensitivity, specificity, positive likelihood ratio, and negative likelihood ratio of the ultrasound for the detection of a moderate-severe fatty liver, as compared to histology, were sufficiently high compared with those of other imaging techniques (i.e., CT or MRI) [1]. Thus, there has been a growing need to have a computer-aided tool to quantify liver steatosis by using the liver echogenicity or the increased US attenuation in fatty liver tissue.

The automated fatty liver diagnosis system typically consists of the detection of the fatty liver area, feature extraction, and classification. The performance of the classifier is highly dependent on the feature set for the classifier algorithms used for the diagnosis. Some of the recent efforts in this line of research are the support vector machine (SVM) with wavelet packet transform (WPT) [9] or gray-level run length matrix (GLRLM) [10], simple neural network, and self-organizing map (SOM) with a textual feature set [11]. Other research efforts in this field include extracting the salient features with the data mining technique [12] or texture analysis [13] or finding an appropriate quantification index to decide a fatty liver class such as the fatty liver index (FLI) [14] and hepatorenal index (HRI) [15].

Further, in practice, several studies report that the ultrasonographic findings of the fatty liver are based on the brightness level of the liver in comparison to the renal parenchyma [16–18]. Since Joseph et al. [19] reported a “bright liver pattern” indicating that a closely packed high amplitude echoes throughout the liver, this pattern has been recognized as a diagnostic hallmark of the fatty liver. Normally, liver and renal cortexes are of a similar echogenicity; however, the renal cortex appears relatively hypoechoic as compared to the liver parenchyma in fatty liver patients on US. The liver-to-kidney contrast has been used as a diagnostic parameter for the fatty liver in many articles [1].

In this study, we also note the importance of the quantification of HRI as a predictor of the fatty liver level and its utility in classification. While many previous studies have viewed the fatty liver classification as a two-class problem (normal versus abnormal), our approach considers it a multiclass problem (normal, mild, moderate, and severe) on the basis of [20].

We also apply a self-organizing map (SOM) and the corresponding cluster analysis in the quantification process of the contrast of HRI with respect to the steatosis level of the liver. SOM has been effectively used in many engineering applications such as computer vision and texture analysis [21] and many areas of medical image analysis [22, 23]. The proposed method does not take into account the quality of the textual feature set that is used in the final fatty liver classification and diagnosis at this point of time. Rather, we focus on the usefulness of SOM and the other corresponding intelligent image processing methods in quantifying HRI with respect to the fatty level of a liver US image.

## 2. Fatty Liver Area Extraction with Image Processing Algorithms

**Figure 1:**
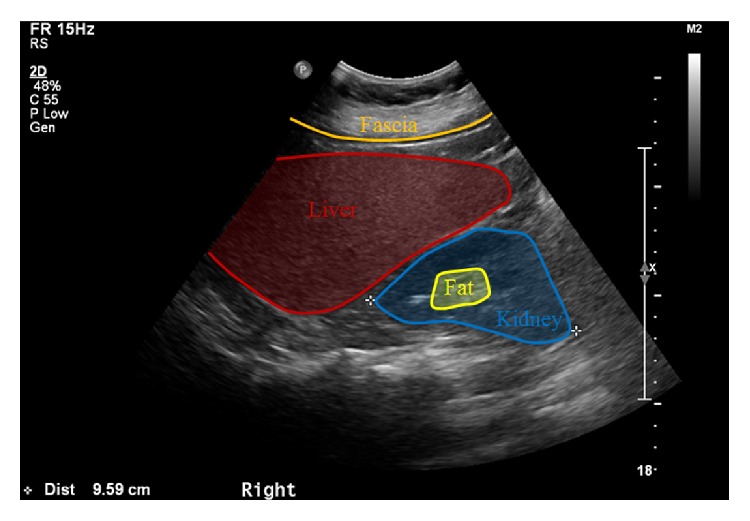
Ultrasonography image and regions of interest.

A typical US image that contains the liver and the right kidney areas and has a relatively low intensity is shown in Figure 1.

There exists a limiting membrane with a high intensity as the border of our two regions of interest (ROI)—the liver and the right kidney. Such ROI are extracted with respect to the location of musculi abdominis and fascia found in the upper area of the liver and the fatty areas of the liver and the kidney.

**Figure 2:**
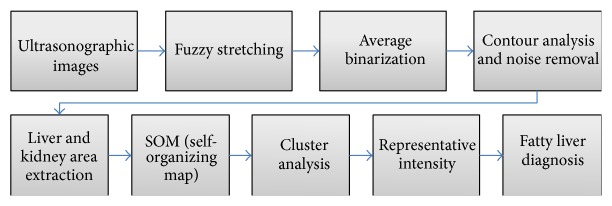
Overall procedure of the proposed method.

Figure 2 summarizes the overall procedures performed in this study. Our software consists of two major activities—ROI extraction with image processing and cluster analysis with an artificial intelligence technique. The latter will be explained in Section 3 in detail.

The first major step of image preprocessing for the US analysis is to enhance the brightness contrast. The US image contains a relatively bright area such as abdomen muscles, fascia, fat area in the kidney, and the border lines between the liver and the kidney. We apply the fuzzy stretching technique [24] for this purpose. The fuzzy stretching technique enhances the contrast by dynamically controlling the maximum and the minimum range of the stretching with a triangle-type fuzzy membership function.

Let *x*_*i*_ be the brightness value of the input US image where *i* is in the range of [0,255]. Then, the average brightness value of the image *x*_*m*_ can be computed by using the following formula:

(1) $$\begin{array}{c} x_{m}=\overset{255}{\underset{i=0}{\sum }}x_{i}\frac{1}{MN}, \end{array}$$

where *M* and *N* denote the width and the length of the image.

Then, the distance between the brighter area and the average *d*_max⁡_, and the darker area and the average *d*_min⁡_ is defined as follows:

(2) dmax⁡xh−xm,dmin⁡=xm−xl,

where *x*_*h*_ and *x*_*l*_ denote the highest and the lowest brightness pixel value, respectively. Then, the brightness values are adjusted according to the following rule and the maximum and minimum intensity values *I*_max⁡_ and *I*_min⁡_ are computed by the following formula:

(3) if  xm>128  ad=255−xmelse  if  xm≤dmin⁡  ad=dmin⁡else  if  xm≤dmax⁡  ad=dmax⁡else  ad=xm,Imax⁡=xm+ad, Imin⁡=xm−ad.

**Figure 3:**
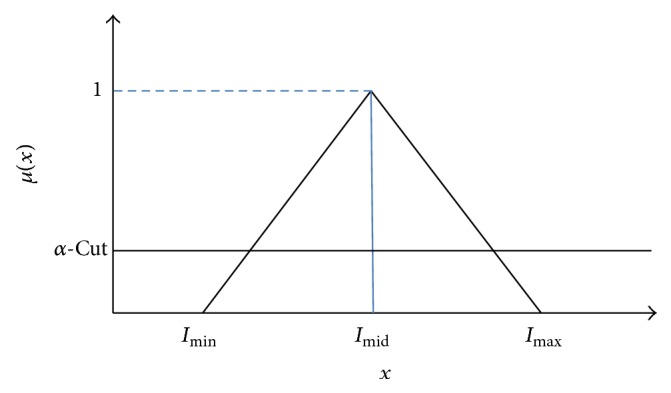
Fuzzy membership function for brightness enhancement.

The fuzzy membership function for intensity stretching is then defined as shown in Figure 3 in the interval [*I*_min⁡_, *I*_max⁡_], where the maximum membership degree *I*_mid_ is defined as follows:

(4) $$\begin{array}{c} I_{\text{mid}}=\frac{I_{\max}+I_{\min}}{2}. \end{array}$$

Thus, the membership degree is defined according to the following rules in the interval [*I*_min⁡_, *I*_max⁡_]:

(5) if  x≤Imin⁡  or  x≥Imax⁡  then  μx=0,if  x>Imid  then  μx=Imax⁡−xImax⁡−Imid,if  x<Imid  then  μx=x−Imin⁡Imid−Imin⁡,if  x=Imid  then  μx=1.

The membership degree *μ*(*x*) obtained from the above rules is then applied to the following formula to compute the lower (*γ*) and the higher (*β*) boundaries of the intensity that are defined as the minimum and maximum values among *x* whose *μ*(*x*) is no less than *α*-cut:

(6) if  Imin⁡≠0  α-cut=Imin⁡Imax⁡else  α-cut=0.5.

**Figure 4:**
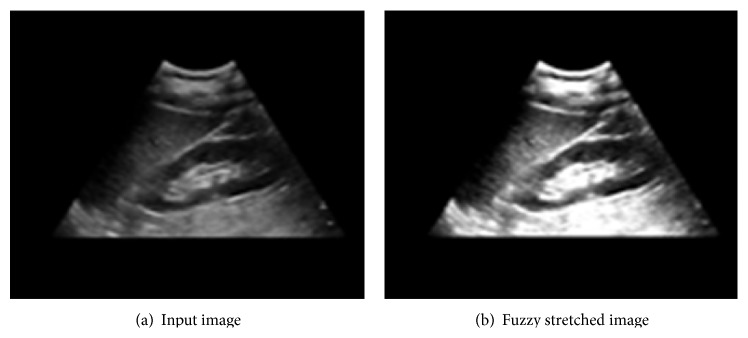
Fuzzy stretching effect.

Finally, the following formula finishes the stretching to enhance the brightness contrast:

(7) $$\begin{array}{c} x^{G}=255\frac{x-\gamma }{\beta -\gamma }, \end{array}$$

where *x* ^*G*^ denotes the stretched brightness value of the pixel from the original value *x*.  Figure 4 demonstrates the effect of fuzzy stretching.

To the image obtained in Figure 4, we also apply average binarization only for pixels with positive intensity.

**Figure 5:**
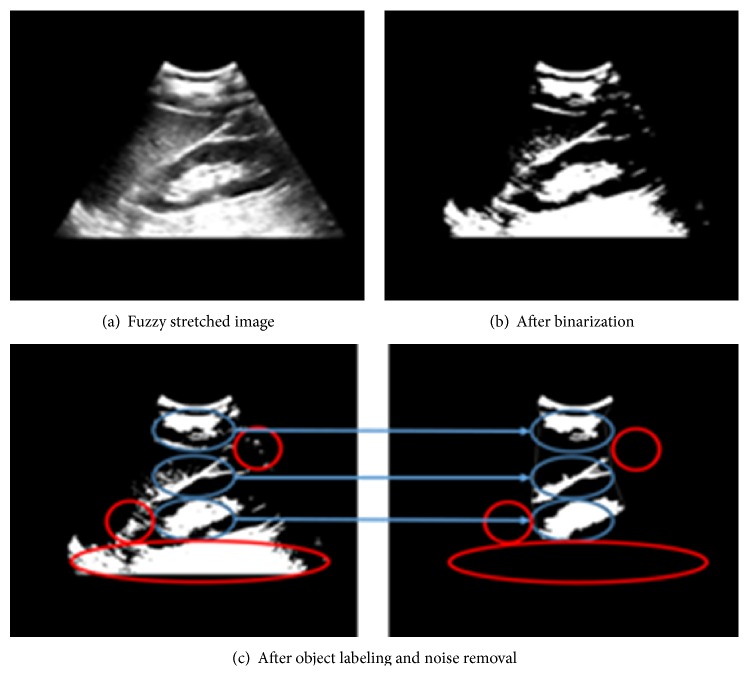
Object extraction by contour analysis.

After binarization, we apply the edge tracking algorithm [25] for fast object labeling. The edge tracking algorithm tracks pixels having the same object label. The search for the same label is 5-directional, starting with the top left spot orthogonal to the current search direction by 45° each until the search point returns to the starting point. If the search diverges or the area obtained by this edge tracking is too small (<200) or too large (>20000), we remove those objects as noise since such a set of objects has no chance to form any fascia, fat in the kidney, or limiting membrane with a high intensity as the border of the liver and the kidney.  Figure 5 demonstrates the effect of binarization (Figure 5(b)) and noise removal by object labeling (Figure 5(c)).

**Figure 6:**
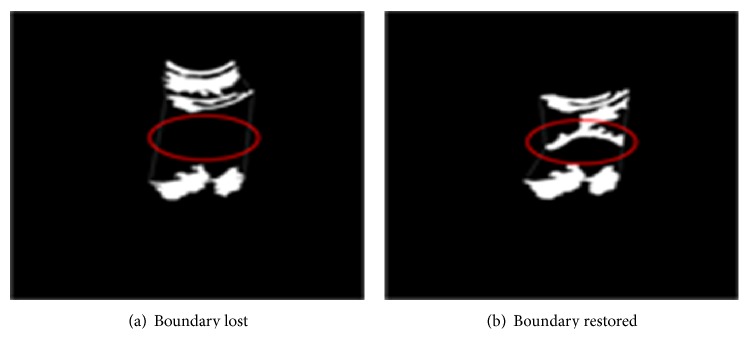
Redoing binarization for boundary line restoration.

However, the limiting membrane, the border between the liver and the kidney, tends to have no clear form with a relatively low intensity; thus, it is easy to lose such information in the brightness-enhancing process. In order to restore such lost boundary lines, we apply the average binarization and labeling procedure again after connecting relatively bright fascia and kidney fat area. This refocusing contour analysis can successfully restore the boundary lines, and the effect can be shown as in Figure 6.

**Figure 7:**
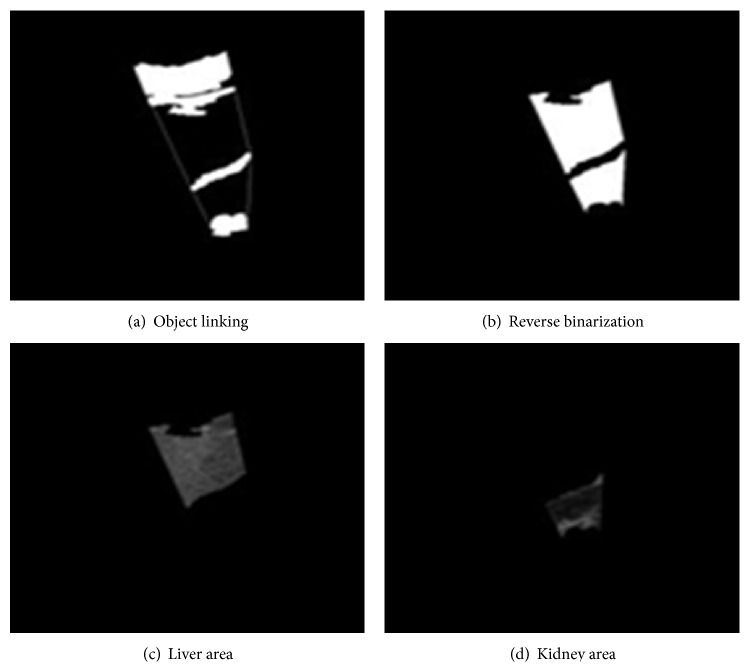
Extracting regions of interest.

The final treatment of this ROI extraction procedure is to discriminate the liver and kidney area with reverse-binarization and AND operations. Knowing that the liver area is located in between the fascia and the limiting membrane boundary lines and the kidney area is located in between the limiting membrane boundary lines and the kidney fat area, we extracted the ROIs as shown in Figure 7 for a further analysis of hepatic steatosis.

## 3. Quantification of Hepatorenal Index by Self-Organizing Map

**Figure 8:**
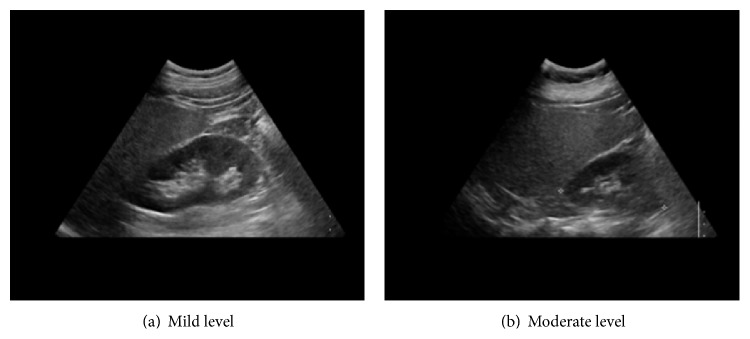
Fatty liver with respect to severity.

In this paper, we adopt the traditional fatty liver classification based on [20] in which abnormal fatty livers are classified into three levels—mild, moderate, and severe—with respect to the evidence of diffuse hyperechogenicity of the liver relative to the kidneys, ultrasound beam attenuation, and poor visualization of the intrahepatic structures.  Figure 8 shows the typical mild and moderate levels of the fatty liver where the mild level (Figure 8(a)) has a slightly increasing echogenicity level of liver parenchyma with clear boundaries of the diaphragm and the intrahepatic blood vessel, whereas the moderate level (Figure 8(b)) has a relatively high increase in the echogenicity and the boundaries become vague. Such a tendency becomes stronger when the fatty liver status is “severe.”

In the US analysis, the brightness level of the liver area with the presence of the right kidney, the hepatorenal index difference (HRI-diff), or hepatorenal index ratio (HRI-ratio) is often used as an index of the abnormal fatty liver classification [15–17]. However, while strongly correlated to other useful classification indexes [18], the quantification of HRI with respect to hepatic steatosis is not a simple problem due to the sensitivity of the probing position and the operator subjectivity.

In this paper, we propose a method based on a self-organizing map (SOM) [21] to quantify HRI-diff automatically. Our goal is to compute the representative brightness value by the cluster analysis obtained by SOM and show that such representative HRI values have a strong statistical tendency with the severity of the fatty liver level.

SOM, a nonlinear, ordered, smooth mapping of high-dimensional data onto the regular, low-dimensional array [21], is an unsupervised learning neural network tool used in many medical image analysis applications [22, 23]. Some studies have used SOM as a diagnostic classifier over a set of textual/statistical features in this fatty liver classification problem [11], but in our work, SOM is used to form a set of stable clusters with respect to the HRI values. Then, the distinction of such clusters with respect to HRI-diff from that of renal parenchyma shows the quantified characteristic of HRI.

We use a two-dimensional output layer in this application, and the SOM algorithm used in this study is as shown in Algorithm 1.

The connection weight (*w*) has the role of sample input patterns and the most similar output neuron *j* becomes the winner during the learning process. Then, all connected weights within the radius *r* from the winner node *j* will be updated. The similarity is computed by the following:

(8) $$\begin{array}{c} D\left(\;j\;\right)=\sum_{i}{\left(\;w_{ji}-x_{i}\;\right)}^{2}. \end{array}$$

Weight (*w* ^*k*+1^) of the learning step *k* + 1 is then defined as follows:

(9) $$\begin{array}{c} \Delta w_{ji}^{k}=\alpha \left[\;x_{i}-w_{ji}^{k}\;\right],\;w_{ji}^{k+1}=w_{ji}^{k}+\alpha \left[\;x_{i}-w_{ji}^{k}\;\right]. \end{array}$$

After the predefined number of repetitions, the radius *r* and the learning rate *α* are reduced and the learning step continues. The stopping condition in our experiment was *α* < 0.2 or radius *r* becomes a nonpositive value.

The clusters from SOM learning are then analyzed after quantization. The quantization process is necessary because of the possible US distractions. Thus, the representative intensity value is defined as the average over pixels in the largest cluster after quantization.

Then, the hepatorenal index difference (HRI-diff) is computed to view if there is a statistically significant tendency with respect to the severity of hepatic steatosis.

Since it is expected that the representative intensity of the kidney area is relatively stable and that of the liver area is positively proportional to the severity of steatosis, HRI-diff will play the role of the predictor of the fatty liver severity classification.

## 4. Experiment and Analysis

The proposed method is implemented in Visual Studio 2010 C# with Intel Core @ 3.40 GHz and 4 GB RAM PC. 25 images from 10 normal, 7 mild, and 8 moderate fatty liver patients were obtained from the Pusan National University Hospital, Korea, in the 1024 × 768 bitmap format. Images were obtained by the right subcostal scan including the lower pole of the liver and the right kidney.  Table 1 summarizes the major findings of this experiment.

As represented in Table 1, HRI values in the liver area are positively proportional to the severity of the hepatic steatosis and even the dispersion of the distribution is clearly discriminative considering low standard deviation within the class level.

**Figure 9:**
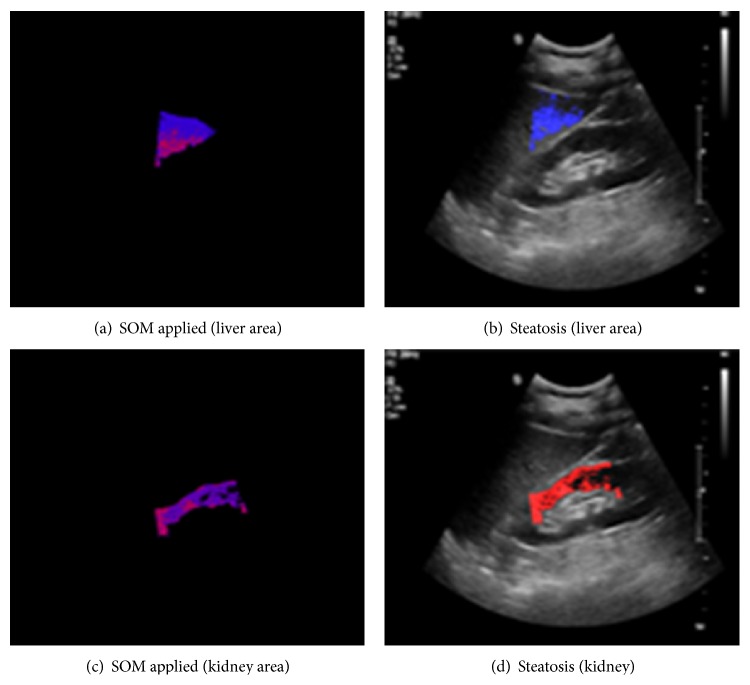
Typical normal liver.

**Figure 10:**
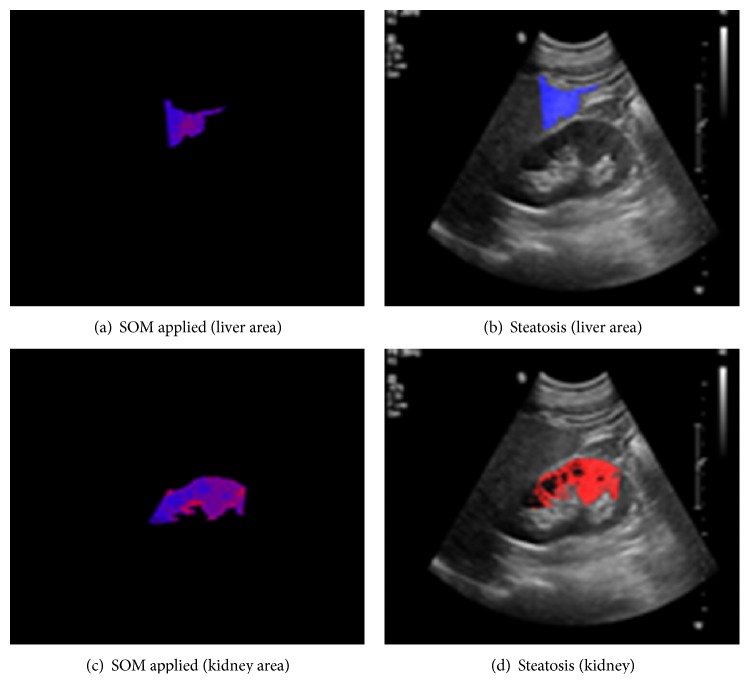
Typical mild fatty liver.

**Figure 11:**
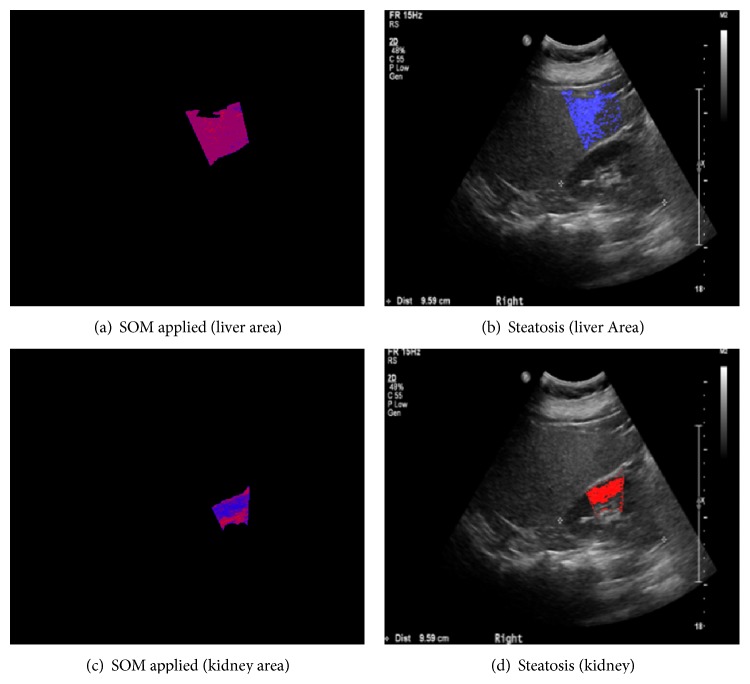
Typical moderate fatty liver.

Figures 9, 10, and 11 show the typical ROI extractions and fatty level visualizations with respect to the severity level—normal, mild, and moderate. At this point of time, we did not have “severe” fatty liver images in this experiment.

## 5. Conclusion

In this study, we aim to quantify the HRI difference between the liver and the kidney by using US images as a useful predictor attribute of the multilevel (multiclass) hepatic steatosis classification. The proposed fully automated computer-aided diagnostic system typically consists of three parts—ROI extraction with image processing, feature set extraction, and applying classifier algorithms. In the ROI extraction procedure, we use a fuzzy stretching algorithm to enhance the brightness contrast. The carefully designed fuzzy membership function and the corresponding auxiliary image processing techniques such as labeling, binarization, and contour analysis enable us to extract the appropriate and distinguishable liver and kidney areas from the image.

Then, the self-organizing map (SOM), an unsupervised neural network learning algorithm, is designed to form representative clusters of the liver image from the ROI US images. The characterization of the cluster analysis gives us a clear statistical delineation of the intensity distribution in terms of HRI-diff among different levels of hepatic steatosis.

The purpose of this study is to show that HRI is an important and informative diagnostic attribute in multiclass fatty liver classification because of such quantification. This encourages us to develop reliable automatic diagnostic software if it is combined with other sets of useful textual or statistical features and other powerful machine learning algorithms in the future.

## Figures and Tables

**Algorithm 1:**
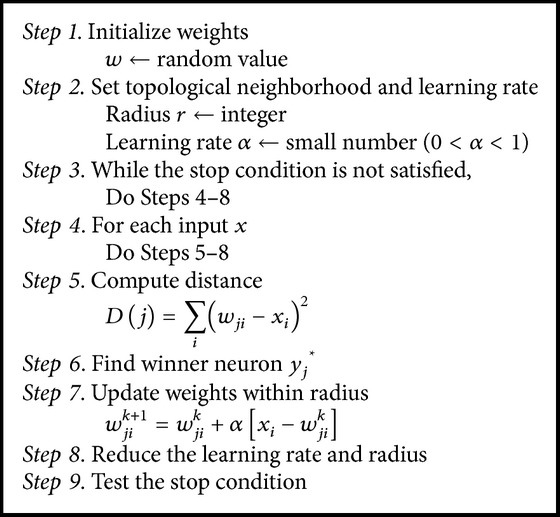
Self-organizing map (SOM) learning algorithm.

**Table 1:** HRI quantification with respect to severity level of fatty liver.

	LP	RP	Number of CL
Normal			
Range	23–53	21–51	8–10
SD	3.027	3.671	
Mean	43.500	41.709	
Mild			
Range	38–74	28–53	9–11
SD	2.546	3.157	
Mean	52.159	43.724	
Moderate			
Range	38–91	28–68	9-10
SD	2.321	4.023	
Mean	68.729	46.704	

LP: liver parenchyma; RP: renal parenchyma; number of CL: number of clusters.
